# N7-methylguanosine-related miRNAs predict hepatocellular carcinoma prognosis and immune therapy

**DOI:** 10.18632/aging.205172

**Published:** 2023-11-03

**Authors:** Liping Ma, Qingwei Ma, Qiaomei Deng, Jilu Zhou, Yingpei Zhou, Qianqian Wei, Zhihu Huang, Xiaoxia Lao, Ping Du

**Affiliations:** 1 Department of Clinical Laboratory, Minzu Hospital of Guangxi Zhuang Autonomous Region, Affiliated Minzu Hospital of Guangxi Medical University, Nanning, Guangxi, China; 2 Department of Gynecology, Minzu Hospital of Guangxi Zhuang Autonomous Region, Affiliated Minzu Hospital of Guangxi Medical University, Nanning, Guangxi, China

**Keywords:** hepatocellular carcinoma, m^7^G, miRNA, prognostic signature, immune therapy

## Abstract

N7-methylguanosine (m^7^G) modification has been notably linked with the development of many tumors. However, no investigations have been conducted on whether m^7^G-related miRNA (m^7^G-miRNA) is a prognostic index of hepatocellular carcinoma (HCC). Therefore, this investigation aimed to establish a predictive m^7^G-miRNA signature for efficient HCC prognosis and elucidate the associated immune cell infiltration (ICI) and functions in the tumor microenvironment. RNA sequencing and clinical data on 375 HCC and 50 healthy tissue samples were acquired from The Cancer Genome Atlas database. The m^7^G-miRNA regulators methyltransferase-like 1 and WD repeat domain 4 were acquired from the TargetScan database. Univariate Cox regression analysis was conducted on the 63 differentially expressed m^7^G-miRNAs identified. A prognostic signature that consisted of seven miRNAs was identified. According to their risk scores, individuals with HCC were divided into high-risk (HR) and low-risk (LR) cohorts. A Kaplan-Meier test revealed that survival in the HR HCC patients was poorer than in the LR cohort (p < 0.001). The area under the receiver operating characteristic curves of 1-, 3-, and 5-year overall survival were 0.706, 0.695, and 0.715, respectively. A nomogram of sex, risk score, age, and stage indicated the HCC patients’ overall survival. Furthermore, it was indicated that the HR and LR patients had different degrees of ICI and immune function. A pathway enrichment analysis revealed the association of several immunity-linked pathways with the risk model. In conclusion, the signature established has great prognostic value and could be used as a new immunotherapy target for individuals with HCC.

## INTRODUCTION

Hepatocellular carcinoma (HCC) is a common liver cancer with a substantial global death rate, ranking as the second most frequent cause of cancer death in men and sixth in women. In 2020, HCC was reported to have afflicted 906,000 new patients and caused 830,000 deaths [1]. HCC is highly malignant, rapidly metastasizes, is mostly detected late, and recurs > 70% of the time after five years of treatment [2]. For accurate HCC diagnosis and prognosis and improved survival time, the identification and validation of prognostic markers and therapeutic targets are urgently required.

N7-methylguanosine (m^7^G) is a conserved RNA modification of the seventh N of RNA guanine. It comprises a methyl group of eukaryotes, prokaryotes, and some archaea. This modification occurs not only in mRNA caps but also at some positions in mRNAs, tRNAs, and rRNAs [3, 4] and is essential for RNA metabolism, including nucleation [5], pre-mRNA splicing [6], transcription elongation [7], and mRNA translation [8]. The literature suggests that m^7^G modification is crucial for regulating biological processes and tumor disease development [9–11]. Aberrant m^7^G expression may affect the incidence and progression of cancer by modulating various tumor suppressors and oncogenes [12]. m^7^G methylation complexes comprise methyltransferase like1 (METTL1) and WD repeat domain 4 (WDR4). According to a study, m^7^G modification and its abovementioned catalyzing enzymes are elevated in HCC cases and linked with a substandard prognosis. Functionally, m^7^G modification promotes HCC progression and tumorigenesis [13]. Nevertheless, the mechanism of m^7^G RNA methylation in HCC is still not fully determined.

MicroRNAs (miRNAs) are small, non-coding, highly conserved RNAs. Although they cannot be translated into protein, they are essential for targeting messenger RNA (mRNA) in gene expression and are thus involved in many biological processes, such as tumorigenesis, progression, and responses to therapy [14, 15]. Individuals with HCC have shown aberrant miRNA levels in multiple studies. Because of their robust presence in body tissues and fluids and easy detectability [16], miRNA expression is not only used as a biomarker for diagnosis and metastasis but also predicts therapeutic response, recurrence, and overall HCC survival rate [17–20]. Research has proven that METTL1 can regulate microRNAs in an m^7^G-dependent pattern. Pandolfini et al. revealed that METTL1 stimulates the processing of let-7 miRNA via m^7^G methylation [21]. METTL1 sensitizes colon cancer cells resistant to cisplatin by modulating miR-149-3p/S100A4/p53 [22]. However, the direct functions of m^7^G and miRNAs in HCC and their associations with HCC, including the molecular markers for predicting HCC prognosis using m^7^G-miRNAs, are still insufficiently understood and require further investigation.

This investigation established a prognostic model based on m^7^G-linked differentially expressed miRNAs (DEmiRNAs) and validated its prognostic and clinical significance. Furthermore, to elucidate the molecular mechanisms dysregulated in HCC, biological pathways and the immune landscape were explored via relevant public databases.

## RESULTS

### Identification of DEmiRNAs

**Figure 1:**
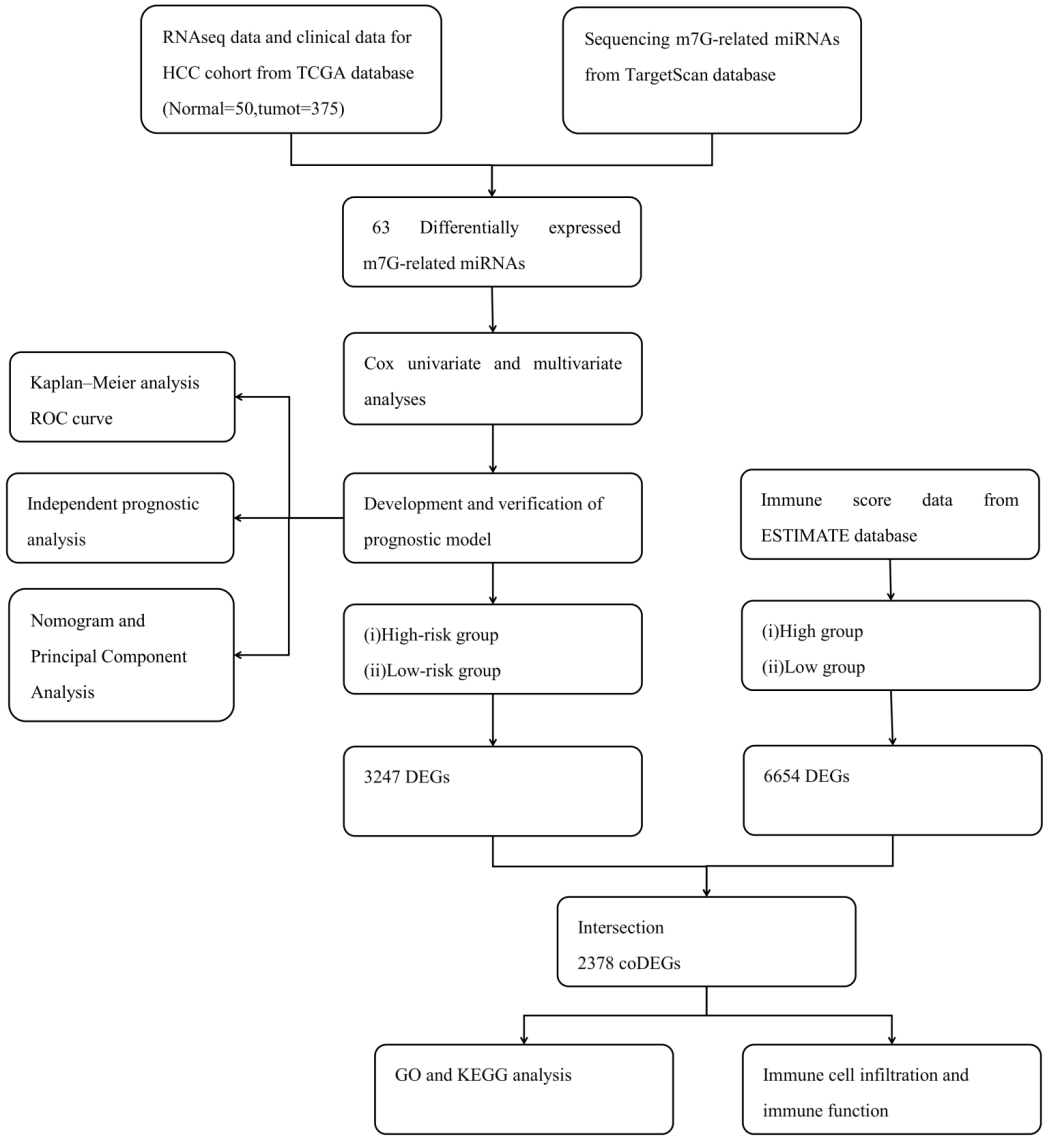
The workflow.

**Figure 2:**
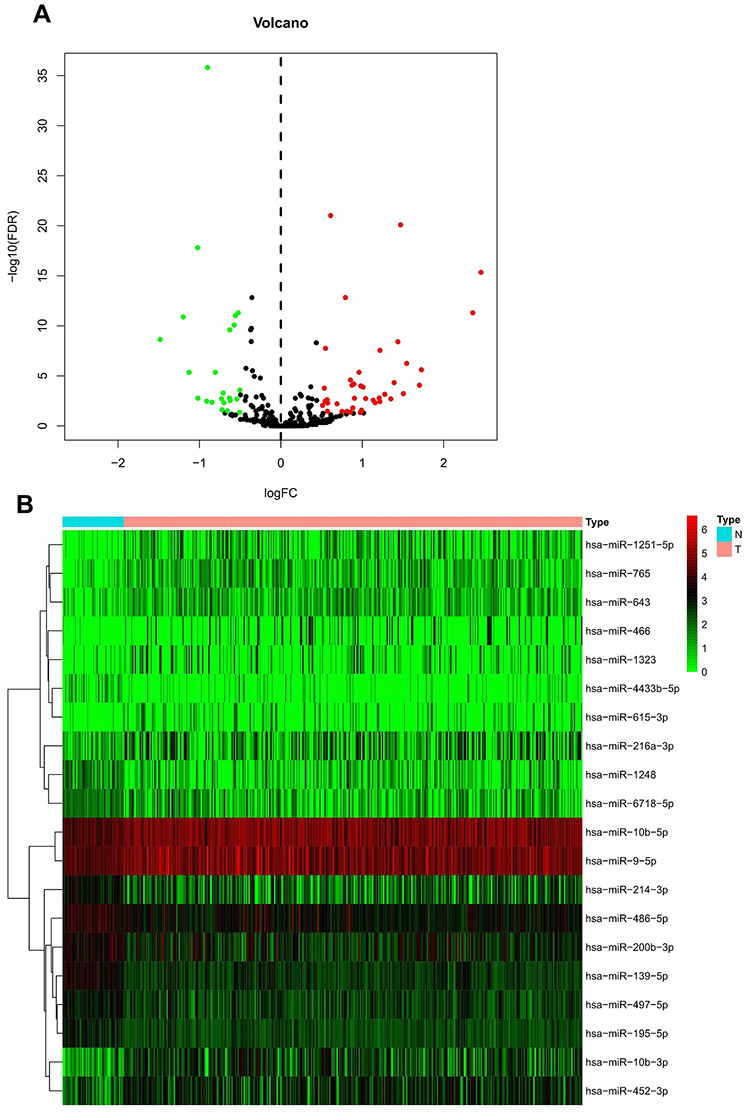
**Differential expression of m^7^G-related miRNA in HCC and non-tumor samples.** (**A**) Heatmap of top 20 DEmiRNAs. Normal and tumor samples are represented in N and T, respectively. (**B**) Volcano plot of all DEmiRNAs. Red and green dots represent up- and down-regulated m^7^G-miRNAs, respectively.

Figure 1 shows the study workflow; 792 miRNAs that targeted WDR4 or METTL1 were identified in TargetScan (Supplementary Table 1). Of these, 63 m^7^G-related miRNAs (m^7^G-miRNAs) were identified as DEmiRNAs in 375 HCC and 50 healthy samples, with 40 defined as up-regulated and 23 as down-regulated according to the cutoff criteria (Figure 2A). The top 20 DEmiRNAs are shown in the heatmap in Figure 2B.

### The m^7^G-miRNA risk model’s generation and validation

**Figure 3:**
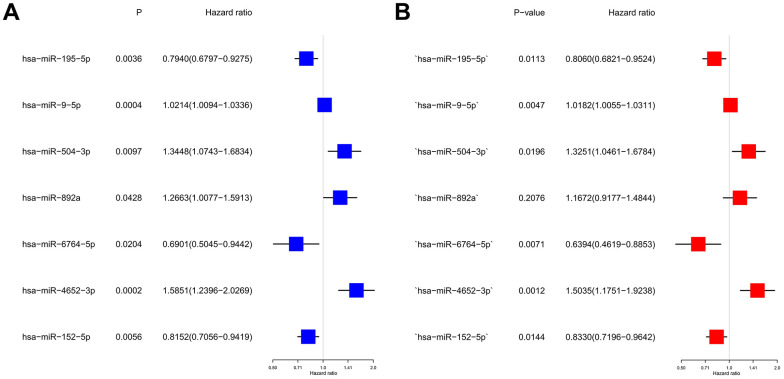
**Construction of risk scores for m^7^G-related miRNAs.** (**A**) Univariate Cox regression analysis was used to screen seven prognosis-related miRNAs. (**B**) Forest plot of multivariate analyses.

**Figure 4:**
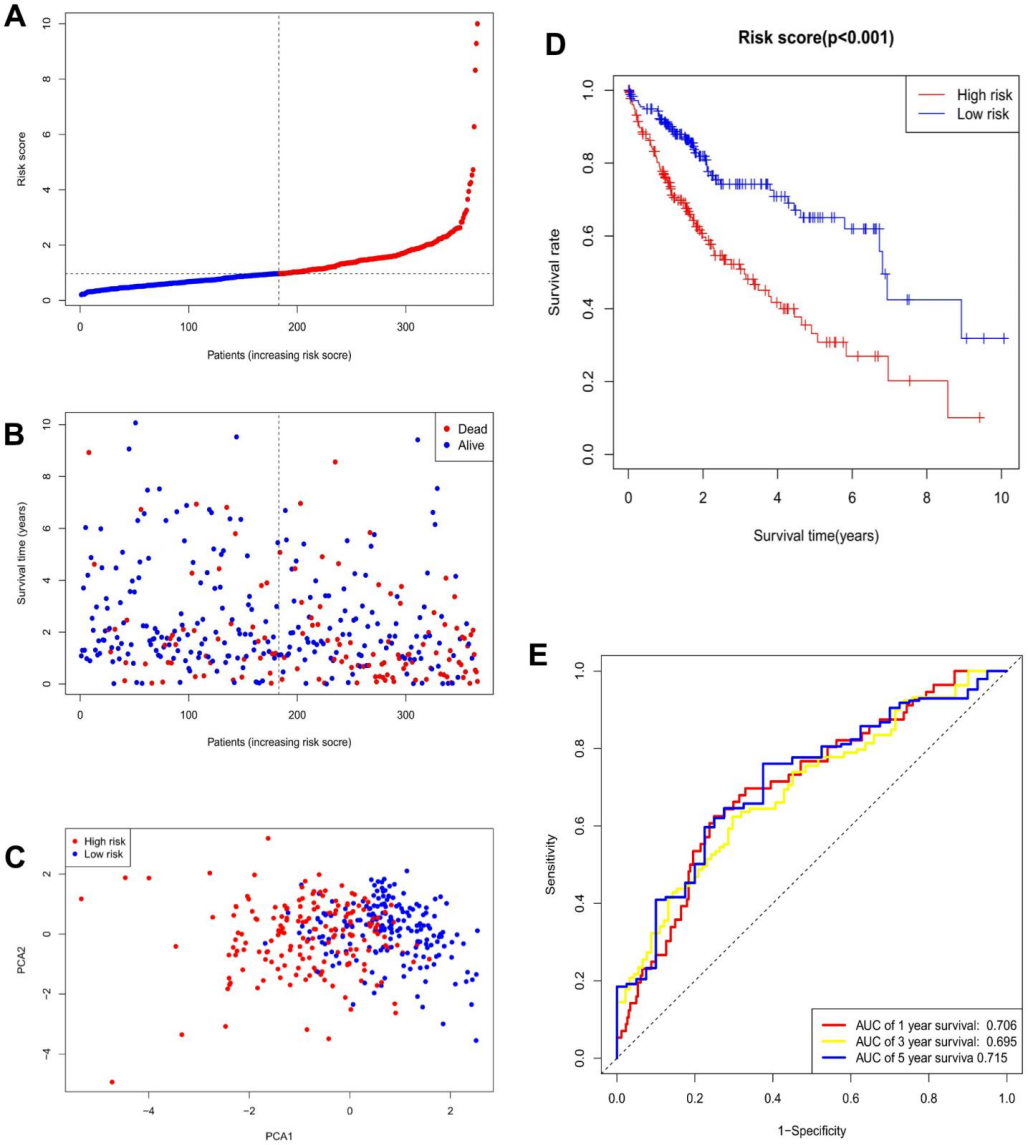
**Validation of prognostic models for seven m^7^G-related miRNAs.** (**A**) Risk score distribution. (**B**) Survival status. (**C**) PCA plot. (**D**) Survival curve for low- and high-risk groups. (**E**) ROC curve for 1-, 3-, and 5-year overall survival.

Based on a univariate Cox regression analysis, seven DEmiRNAs (hsa-miR-195-5p, hsa-miR-9-5p, hsa-miR-504-3p, has-miR-892a, hsa-miR-6764-5p, hsa-miR-4652-3p, hsa-miR-152-5p) were selected for use in constructing a prognostic risk regression model (Figure 3A, 3B). A total of 366 HCC case records with survival time data were used to construct and verify this model. Risk scores were determined for all subjects, and the latter were divided into two groups according to the risk score median. The low-risk (LR) and high-risk (HR) score cohorts included 183 patients each (Figure 4A). It is clear from Figure 4B that the mortality rate in the HR group was significantly higher than that in the LR group, which indicates valid sample grouping. The grouping’s accuracy was also ensured using principal component analysis (PCA), the results of which show that the HCC cases in the LR and HR groups were clearly separated (Figure 4C). According to a Kaplan–Meier survival test, the HR HCC patients’ overall survival rate was poorer than that of the LR patients (Figure 4D). The risk model’s predictive ability was evaluated by time receiver operating characteristic (ROC) analysis; at 1, 3, and 5 years, the areas under the curve (AUCs) of OS were 0.706, 0.695, and 0.715 (Figure 4E). This result indicates that the risk model has moderate diagnostic strength.

### Independent prognostic factors of the final model

**Figure 5:**
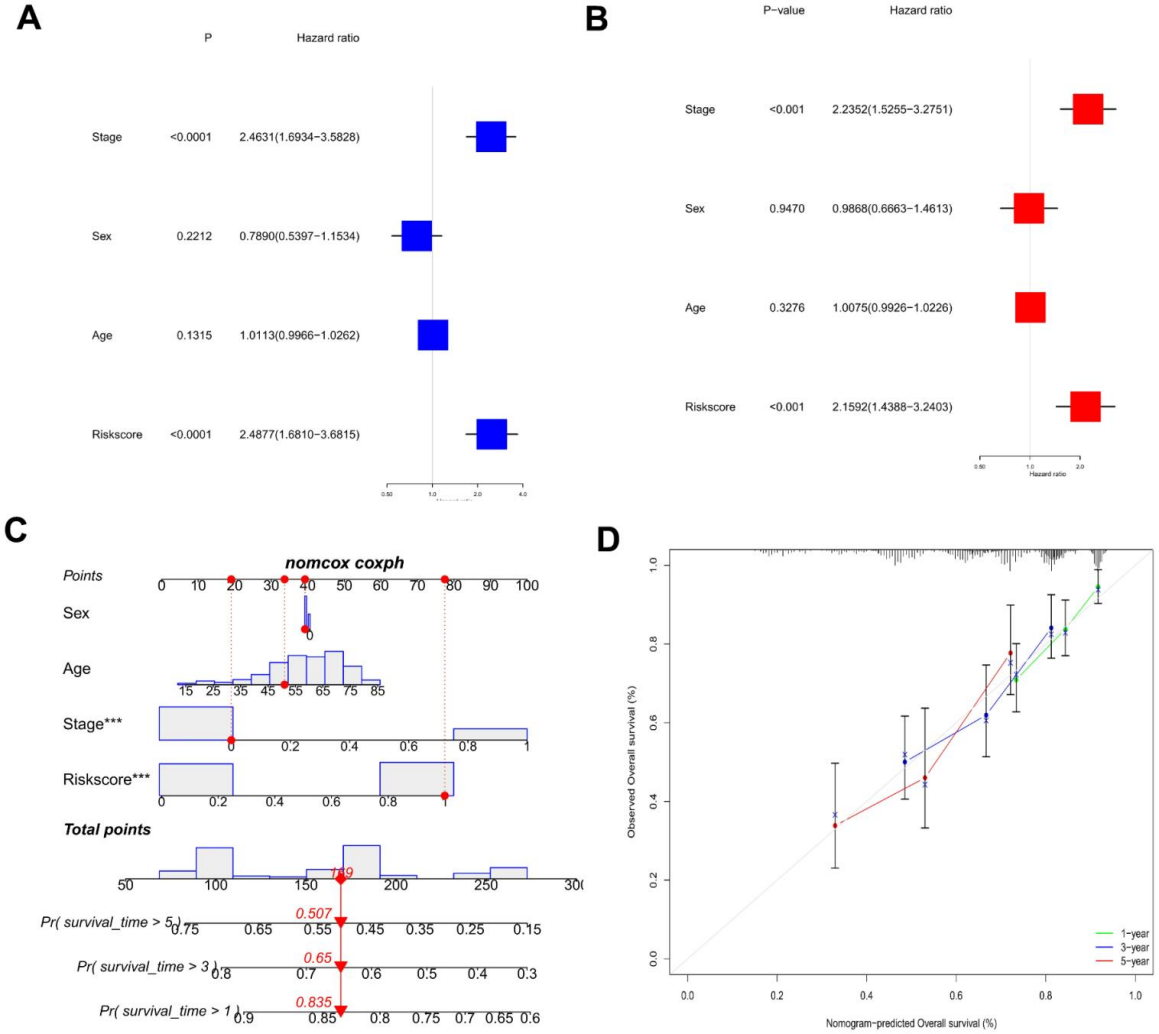
**Establishment of a predictive nomogram.** (**A**) Univariate Cox regression analysis of risk scores and clinical characteristics in HCC samples. (**B**) Multivariate Cox regression analysis of risk scores and clinical characteristics in HCC samples. (**C**) Nomogram used to predict the survival of the HCC patients. (**D**) Calibration curve for 1-, 3-, and 5-year overall survival.

The risk scores were combined with the clinical parameters to perform univariate and multivariate Cox regression analyses to elucidate the independent predictive potential of this risk model. Risk score and stage were indicated to be independent predictors (Figure 5A, 5B). A univariate Cox regression analysis (p < 0.001) of risk score [hazard ratio (HR) = 2.488 and 95% confidence interval (CI) = 1.681–3.682] and stage [HR = 2.463 and 95% CI = 1.693–3.583] as well as a multivariate Cox regression analysis (p < 0.001) of risk score [HR = 2.159 and 95% CI = 1.439–3.240] and stage [HR = 2.235 and 95% CI = 1.526–3.275] were performed. Furthermore, a nomogram was established for risk measurement and clinical variables (stage, sex, age) to predict HCC patient outcomes (Figure 5C). Based on the calibration curve, the nomogram’s predictive performance was good (Figure 5D).

### Association of miRNA candidates with HCC patients’ survival

**Figure 6:**
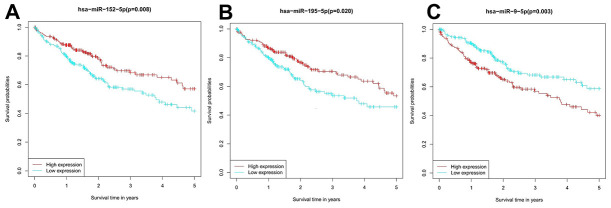
**Survival analysis of the prognostic value of the miRNA candidates in HCC patients.** (**A**) hsa-miR-195-5p, (**B**) hsa-miR-152-5p, and (**C**) hsa-miR-9-5p.

Survival analysis was performed to evaluate the prognostic value of the candidate miRNAs for HCC. Patients with high hsa-miR-195-5p and hsa-miR-152-5p expression had a notably higher survival rate than patients with reduced expression, while those with high hsa-miR-9-5p expression showed a markedly lower survival rate (Figure 6).

### Enrichment analyses

**Figure 7:**
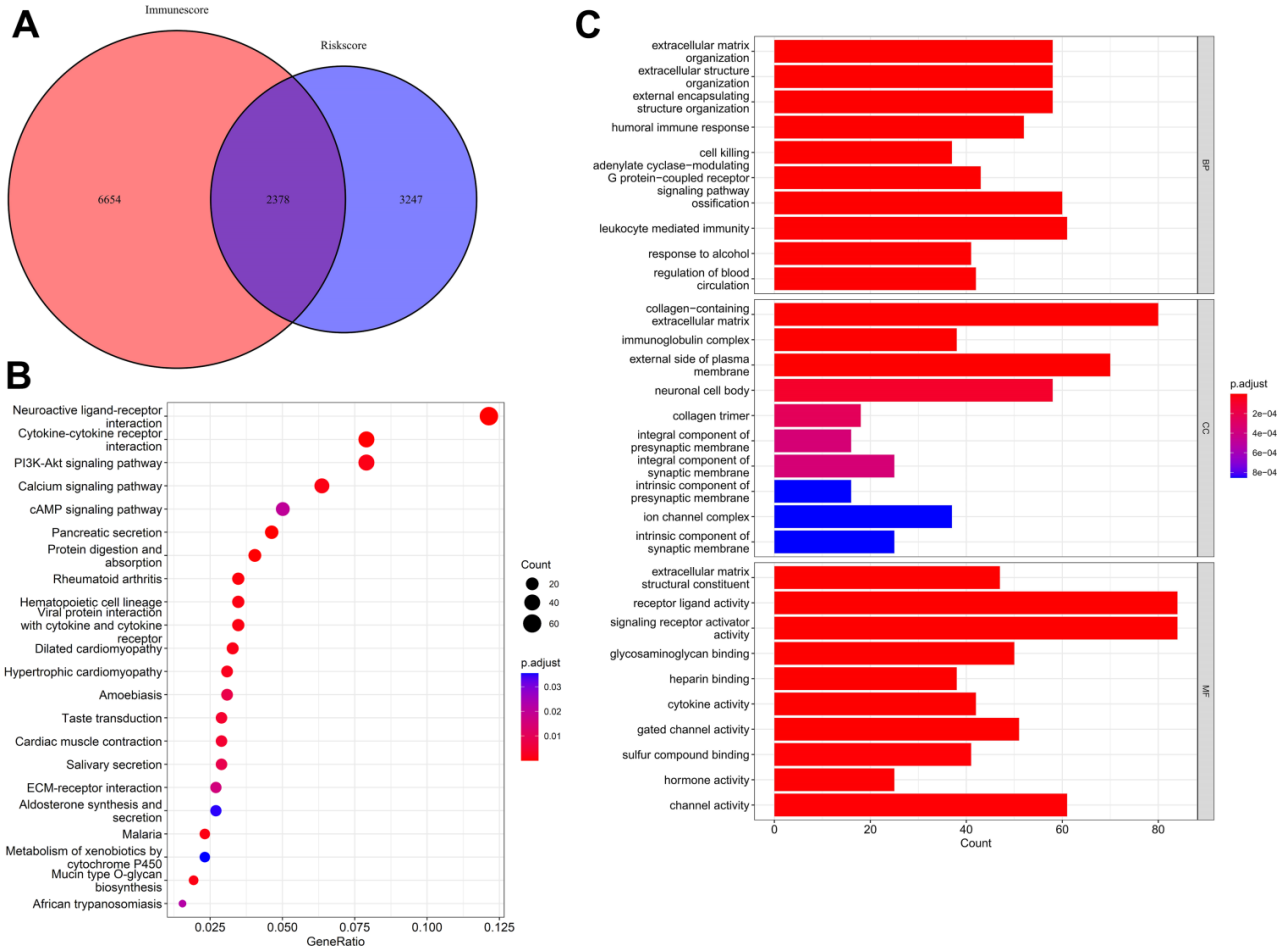
**Identification and functional enrichment analysis of coDEGs.** (**A**) A total of 2,378 coDEGs were obtained by finding the intersections. (**B**) KEGG enrichment analysis shown in dot plots. (**C**) GO enrichment analysis shown in bar plots.

A differential expression analysis identified 3,247 differentially expressed genes (DEGs) after comparing the LR and HR cohorts and 6,654 DEGs after comparing high immune score (HIS) and low immune score (LIS) cohorts. As the Venn diagram depicts, 2,378 overlapping DEGs were identified and considered co-differentially expressed genes (coDEGs) (Figure 7A). Gene Ontology (GO) and Kyoto Encyclopedia of Genes and Genomes (KEGG) enrichment analyses uncovered the possible functions and molecular mechanisms of these coDEGs. The KEGG analysis revealed the top three pathways, namely the cytokine–cytokine receptor interaction, PI3K-Akt signaling pathway, and neuroactive ligand–receptor interaction (Figure 7B). The GO analysis indicated that the biological processes (BP) of the coDEGs were primarily enriched in leukocyte-regulated immunity, ossification, and extracellular matrix organization. The cellular components (CC) include an extracellular matrix containing collagen, the plasma membrane’s external side, and the neuronal cell body. The molecular functions (MF) include receptor ligand, signaling receptor activator, and channel activity (Figure 7C).

### Immune infiltration in the tumor microenvironment

**Figure 8:**
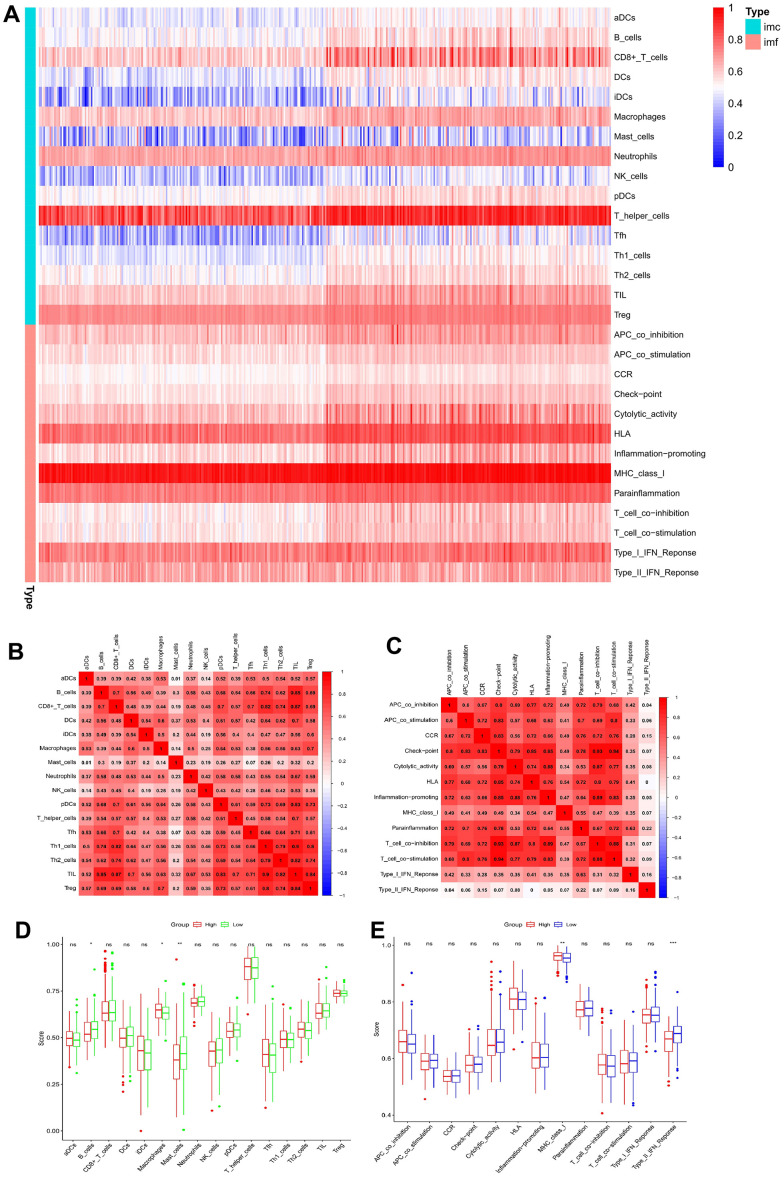
**Correlation analysis between ssGSEA scores and immune cells or immune functions.** (**A**) Heatmap of immune infiltration based on ssGSEA. Immune cells and immune functions are represented by imc and imf, respectively. (**B**) Correlation analysis of various immune cells. (**C**) Correlation analysis of various immune functions. (**D**) Box plots for comparing the ssGSEA scores of various immune cells in the high-risk and low-risk groups. (**E**) Box plots for comparing the ssGSEA scores of various immune functions in the high-risk and low-risk groups (*p < 0.05, **p < 0.01, and ***p < 0.001; ns: no significance).

A single sample gene set enrichment analysis (ssGSEA) revealed different degrees of 28 immune cell infiltration (ICI) and 13 immune functions in the HCC samples. In Figure 8A, a heatmap of immune infiltration based on the ssGSEA scores demonstrates the prominent expression of major histocompatibility complex class 1 (MHC class I), T helper cells, human leukocyte antigen, a type I interferon response, and parainflammation in the tumor immune microenvironment of HCC patients.

Spearman correlation was used to assess the associations between various immune cells and immune functions in the immune microenvironment of HCC cases. The closer the correlation coefficient is to 1, the higher the correlation of immune cells or immunity functions. For ICI, Th1 cells and tumor-infiltrating lymphocytes showed the highest correlation, with an r-value of 0.90 (Figure 8B). Among the various immunity functions, the correlation between immune checkpoint and T-cell co-stimulation was the highest (r = 0.94), followed by that between immune checkpoint and T-cell co-inhibition (r = 0.93) (Figure 8C).

**Figure 9:**
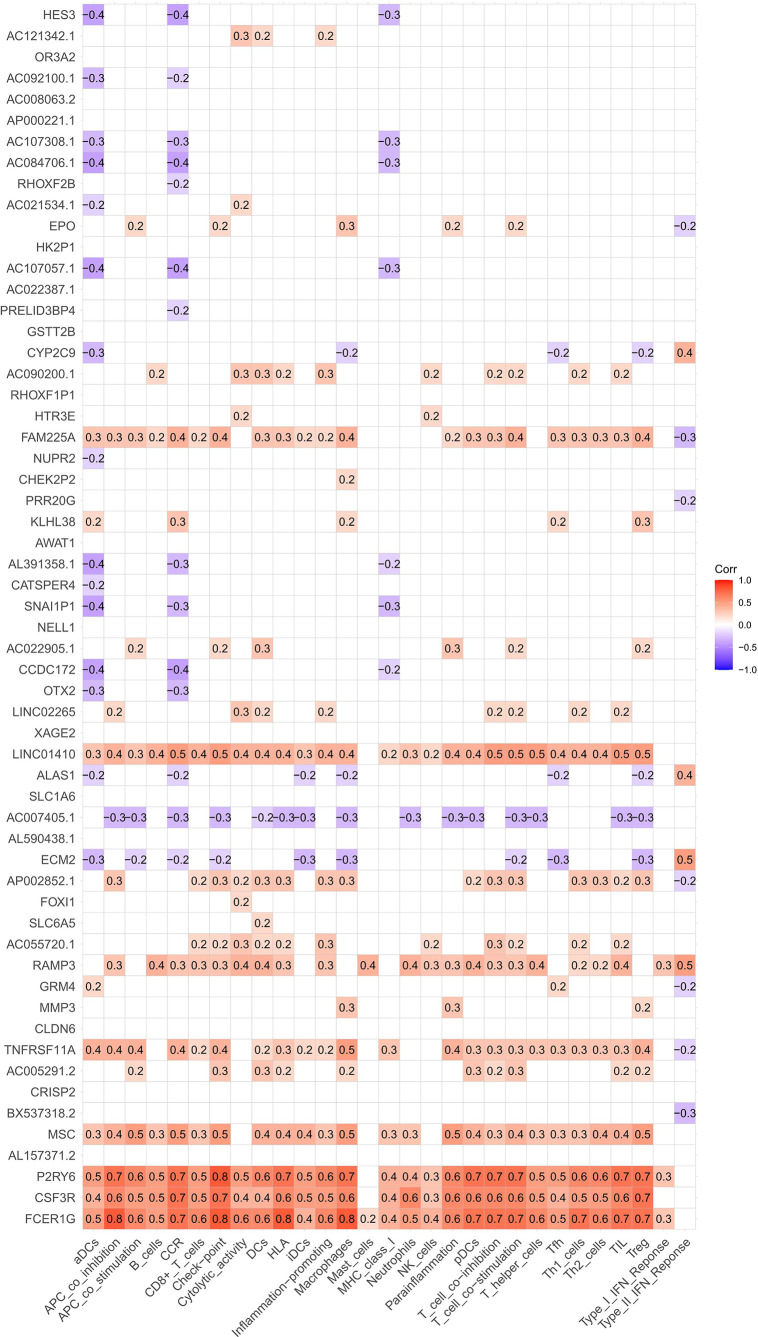
Correlation analysis of coDEG expression with immune cell infiltration and functions.

Furthermore, the ssGSEA scores of immune cells and functions in the HCC cohort were compared, indicating that the proportions of B and mast cells were substantially reduced and that those of macrophages were higher in the HR cohort than in the LR cohort (Figure 8D). In the immune functions, only type II interferon indicated a greater response in an HR cohort (Figure 8E). Finally, the association between the coDEGs’ expression and 16 different immune cell subtypes or 13 immune functions revealed that most coDEG expressions were positively linked with the degree of ICI and immune functions (Figure 9).

## DISCUSSION

Currently, various miRNA signatures can accurately predict disease prognoses, as when a signature comprising 11 miRNAs was used to forecast an endometrial cancer prognosis [23]. A necroptotic miRNA-based signature can predict colon cancer patients’ overall survival rate [24]. Furthermore, m^7^G-miRNA-based signatures have been reported in tumor research [25]. For instance, Hong et al. constructed a risk model including seven m^7^G-miRNAs in kidney renal clear cell carcinoma that could be used to predict patient prognosis and personalize immunotherapy [26]. Xu et al. developed an m^7^G-miRNA signature to predict the overall survival rate and immune landscape of triple-negative breast cancer [27]. However, the systematic analysis of m^7^G-miRNA signatures to predict HCC survival has not been undertaken. Therefore, this investigation established an m^7^G-miRNA-based signature to predict HCC sufferers’ survival. Additionally, the variations in the risk models in terms of ICI and immune function were determined.

The TargetScan database was used to search for m^7^G-miRNAs, and seven miRNAs were identified for use in constructing a novel prognostic signature for HCC: hsa-miR-195-5p, hsa-miR-9-5p, hsa-miR-504-3p, has-miR-892a, hsa-miR-6764-5p, hsa-miR-4652-3p, and hsa-miR-152-5p. The literature suggests that these miRNAs are notably linked with cancer pathogenesis. miR-195–5p expression is frequently downregulated in numerous tumors, including osteosarcoma [28], prostate cancer [29], and HCC [30]. miR-195–5p acts as a tumor suppressor by targeting various oncogenes, such as Fos-like antigen-1, basic fibroblast growth factor, programmed death-ligand 1, axin2, and myc binding protein [31]. Accordingly, miR-195–5p reportedly involves various tumorigenesis and developmental processes, such as migration, apoptosis, invasion, proliferation, and chemoresistance [32–34]. Therefore, investigating miR-195–5p might reveal diagnostic indices and therapeutic cancer targets [35]. miR-9-5p was aberrant expressed in various tumor tissues and developed different functions by targeting different mRNAs. For instance, miR-9-5p functions as an oncomiR, acting against curcumin and paclitaxels’ cytotoxic synergistic effects by inhibiting BRCA1 in ovarian cancer cases [36]. However, it has also acted as a tumor suppressor by inhibiting tumorigenesis and chemosensitivity by targeting neuropilin-1 in gastric cancer cases [37]. It was reported that miR-504-3p suppresses tumors’ malignant phenotypes by reducing interferon-induced transmembrane protein 1 or methylenetetrahydrofolate dehydrogenase 2 expression [38, 39]. In gastric cancer cases, it was found that miR-892a overexpression predicts a substandard prognosis and clinical pathological characteristic [40]. lncRNA CAR10 regulates the expression of gap junction protein beta 2 via miR-892a, thus promoting the migration and invasion capacity of non-small-cell lung cancer cells [41]. Dysregulation of miR-6764-5p was identified in pituitary adenoma cases [42]. Spen family transcriptional repressors activate PI3K/AKT signaling to modulate the c-JUN/miR-4652-3p/HIPK2 axis, thereby activating epithelial-mesenchymal transition signaling and promoting nasopharyngeal carcinoma metastasis [43]. In malignant meningioma cases, miR-4652-3p has been associated with LINC00702-regulated Wnt/β-catenin signaling and attenuated the cell proliferation and migration caused by this pathway [44]. miR-152-5p inhibits malignant progression and tumorigenesis by targeting FBXL7, potentiating gliomas’ temozolomide sensitivity [45]. Furthermore, miR-152-5p has been reported to suppress tumors from gastric cancer by targeting PIK3CA [46]. miR-152-5p overexpression enhances the expression of apoptosis-related factors and forkhead transcription factor O by activating the JNK pathway and inhibiting malignant liver cancer phenotypes [47].

In this study, risk-score-based LR and HR cohorts were used to establish a signature that could predict HCC patients’ OS with high accuracy, as evidenced by survival analysis. Furthermore, univariate and multivariate Cox regression analyses revealed that risk score was markedly linked with HCC OS. According to the PCA, the model differentiated the LR and HR cohorts efficiently. This suggests the reliability of the seven m^7^G-miRNAs mentioned above in predicting the OS of HCC patients.

HCC is induced by a chronic inflammatory state at its initiation and development. To understand the importance of the infiltration of various immune cells and alterations in immune function linked with HCC in the LR and HR cohorts, ssGSEA was applied to evaluate the differential immune scores. It has been suggested that tumor-infiltrating macrophages of the alternatively activated macrophage (M2) phenotype have pro-inflammatory and tumor-promoting effects by suppressing anti-tumor immune response [48]. Increased infiltration of M2 yielded a poorer prognosis [49]. Consistent with this study, an association between high macrophage infiltration and substandard HCC progression was observed. In addition, increased type II IFN response was observed in the LR cohort. These are essential components of immune response against infections and cancers, stimulating pro-inflammatory responses crucial for immune activation and inducing immune-repressing feedback circuits to inhibit cancer growth [50]. This indicates that immunotherapy is more effective in HR than in LR cohorts. Thus, an m^7^G-miRNA-based signature may provide potential clues for developing HCC immunotherapy. Furthermore, 2,378 coDEGs were identified for GO and KEGG enrichment analyses based on the signatures’ risk and immune scores. The coDEGs were primarily enriched in humoral immune response, cell killing, leukocyte-mediated immunity, and cytokine–cytokine receptor interaction. The association of coDEG expression with ICI and immune function revealed a correlation that was more positive than negative. All these results suggest that m^7^G-miRNA may affect HCC prognosis via immune mechanisms.

### Limitations

The limitations of this investigation were as follows: 1) The conclusions drawn are based on integrative bioinformatics and experimental verification. Further study of the HCC cohort is required to validate these conclusions. 2) Functional experiments illustrating how these m^7^G-miRNAs affect HCC are missing. 3) The m^7^G-miRNA signature’s accuracy in HCC prognosis and immune regulation is crucial for clinical studies, and clinical findings are required to further validate this prognostic model.

## CONCLUSIONS

An m^7^G-miRNA prognostic model with a substantial capacity to predict OS in HCC was established. Risk score’s influence on ICI and immune function was elucidated. Moreover, a functional analysis of coDEGs and their relationships with ICI and immune function was provided. As far as we know, this is the first m^7^G-miRNA-based prognostic model for HCC and could, therefore, help us discover novel therapeutic HCC targets.

## MATERIALS AND METHODS

### Data collection

The clinical and RNA-seq data of 375 HCC and 50 healthy tissues were acquired from The Cancer Genome Atlas (TCGA) database in May 2023. The subsequent analyses were performed using the m^7^G methylation complexes METTL1 and WDR4.

### Constructing the m^7^G-related miRNA HCC-prognosis prediction model

m^7^G-related miRNAs (m^7^G-miRNAs) were identified via the TargetScan e-database (https://www.targetscan.org/vert_72/). DEmiRNAs were screened with the help of the “edgeR” package in R, with a false discovery rate < 0.05 and |log [fold change] | ≥ 0.5. Heat maps were drawn using the “heatmap” package.

With the help of univariate Cox regression analysis, prognostic miRNAs were screened using the “survival” package, with p < 0.05. Seven candidate miRNAs were identified and used for the multivariate Cox regression model. Each patient’s risk score was assessed as follows:

risk score = ∑ Coef miRNA × log 2 (miRNA expression + 1).

According to the risk score median, the HCC samples were categorized into HR and LR cohorts, and their OS difference was evaluated using Kaplan–Meier analysis. The prognostic model’s sensitivity and specificity were elucidated using the ROC curve with the AUC using the “time ROC” package in R. Finally, the HCC samples’ clinical variables (including age, pathological stage, and sex) and the risk score for Cox (univariate and multivariate) regression analyses demonstrated independent prognostic risk score indicators. This completed the prognostic risk model based on the seven miRNA candidates.

### Predictive nomogram construction and principal component analysis

The nomogram and calibration curves predicted 1-, 3-, and 5-year survival rates. PCA was carried out using the “scatterplot3D” R package to elucidate the number of individuals with HR and LR scores.

### Survival analysis of prognosis-relevant miRNAs

A survival analysis was conducted to explore the correlation between the expression of prognosis-relevant miRNAs and prognoses in HCC patients. HCC patients were categorized as high- and low-expression patients according to the median expression level of each miRNA. Patients whose miRNA expression level was above the median expression level of this gene were defined as high-expression patients; otherwise, they were low-expression patients. A Kaplan–Meier survival curve was drawn using the “survival” package in R, and the OS figures were compared using a log rank test.

### Screening of differentially expressed genes

The HCC cohort’s immune scores were obtained via the “estimation of stromal and immune cells in malignant tumor tissues using expression data” method. On the basis of the immune score median, HCC individuals were categorized into HIS and LIS cohorts. The DEGs of different subgroups (HR vs. LR score and HIS vs. LIS) were screened using the “edgeR” package, and the intersecting points were acquired as coDEGs. The cutoff values were based on a |log (FC) | ≥ 0.5 and FDR < 0.05.

### Enrichment analyses

To elucidate the primary biological processes and coDEG pathways, GO and KEGG enrichment assays were conducted using the “ClusterProfiler” package.

### Immune infiltrate analysis

With the help of the “GSVA” package, ssGSEA scores were used to quantify ICI and immune function in HCC. Furthermore, the correlation between different ICI statuses and immune functions was assessed using Spearman analysis. ICI statuses and immune functions in the LR and HR cohorts were compared via the “ggpubr” package. Additionally, the association of coDEGs with ICI statuses and immune functions was evaluated.

### Statistical measurements

The data were measured statistically using RStudio (version 4.0.4) and its packages (limma, ggplot2, survminer, timeROC, GSVA, etc.). Cox univariate and multivariate analyses were used to construct a prognosis-prediction model and evaluate the independent prognostic value of the clinical characteristics of OS. Kaplan–Meier analysis was used to compare OS in the various subgroups. The prognostic ability of the predictive models was evaluated based on the area under the ROC curve. Student’s t-test was used to compare the two groups’ differences. Spearman’s correlation was used to calculate the association between different ICI statuses and immune functions. A two-tailed P < 0.05 was considered to indicate statistical significance.

### Data availability statement

The datasets presented in this study can be found in online repositories.

## Supplementary Material

Supplementary Table 1
